# Proximity Elongation Assay and ELISA for the Identification of Serum Diagnostic Biomarkers in Parkinson’s Disease and Progressive Supranuclear Palsy

**DOI:** 10.3390/ijms252111663

**Published:** 2024-10-30

**Authors:** Costanza Maria Cristiani, Camilla Calomino, Luana Scaramuzzino, Maria Stella Murfuni, Elvira Immacolata Parrotta, Maria Giovanna Bianco, Giovanni Cuda, Aldo Quattrone, Andrea Quattrone

**Affiliations:** 1 Neuroscience Research Center, University “Magna Graecia”, 88100 Catanzaro, Italy; 2 Research Centre for Advanced Biochemistry and Molecular Biology, Department of Clinical and Experimental Medicine, University “Magna Graecia”, 88100 Catanzaro, Italy; 3 Institute of Molecular Biology, Department of Medical and Surgical Sciences, University “Magna Graecia”, 88100 Catanzaro, Italy; 4 Institute of Neurology, Department of Medical and Surgical Sciences, University “Magna Graecia”, 88100 Catanzaro, Italy

**Keywords:** Parkinson’s disease, progressive supranuclear palsy, PEA, machine learning

## Abstract

Clinical differentiation of progressive supranuclear palsy (PSP) from Parkinson’s disease (PD) is challenging due to overlapping phenotypes and late onset of PSP specific symptoms, highlighting the need for easily assessable biomarkers. We used proximity elongation assay (PEA) to analyze 460 proteins in serum samples from 46 PD, 30 PSP patients, and 24 healthy controls. ANCOVA was used to identify the most promising proteins and machine learning (ML) XGBoost and random forest algorithms to assess their classification performance. Promising proteins were also quantified by ELISA. Moreover, correlations between serum biomarkers and biological and clinical features were investigated. We identified five proteins (TFF3, CPB1, OPG, CNTN1, TIMP4) showing different levels between PSP and PD, which achieved good performance (AUC: 0.892) when combined by ML. On the other hand, when the three most significant biomarkers (TFF3, CPB1 and OPG) were analyzed by ELISA, there was no difference between groups. Serum levels of TFF3 positively correlated with age in all subjects’ groups, while for OPG and CPB1 such a correlation occurred in PSP patients only. Moreover, CPB1 positively correlated with disease severity in PD, while no correlations were observed in the PSP group. Overall, we identified CPB1 correlating with PD severity, which may support clinical staging of PD. In addition, our results showing discrepancy between PEA and ELISA technology suggest that caution should be used when translating proteomic findings into clinical practice.

## 1. Introduction

Parkinson’s disease (PD) and progressive supranuclear palsy (PSP) are two neurodegenerative parkinsonian syndromes, both characterized by bradykinesia, rigidity, and postural and gait impairment [1,2]. Some specific symptoms, such as supranuclear gaze palsy, may guide the differential diagnosis when patients develop the full clinical picture [1,2]; however, the overlap in clinical signs may cause a high rate of misdiagnosis, especially in the early stages of the diseases [1,2,3]. For this reason, significant research has been devoted to the identification of biomarkers that could support the clinical diagnosis. From a molecular point of view, PD is a synucleinopathy [4] while PSP is a tauopathy [5]; thus, α-synuclein and tau proteins have been investigated as diagnostic biomarkers in cerebrospinal fluid (CSF) and, more recently, in more accessible fluids such as plasma and serum [6]. However, results from these hypothesis-driven biomarkers were inconclusive [7,8,9,10,11,12,13,14,15], suggesting that unbiased analyses might be more informative.

Quantitative proteomics allows the simultaneous screening of a wide range of analytes to identify protein signatures. Among the different techniques, OLINK proximity elongation assay (PEA) is increasingly used in parkinsonism research to identify diagnostic and prognostic biomarkers [16,17,18,19,20,21,22]. OLINK is a 96-plex immunoassay employing antibody binding and oligonucleotide elongation through quantitative real-time polymerase chain reaction (qPCR) for the high-throughput identification and quantification of proteins within biofluids [22]. However, OLINK is a complex methodology hardly applicable in clinical practice, where simpler assays and more immediate results are highly preferred. In addition, although CSF is a significant biological sample for neurodegenerative disorder, lumbar puncture is an invasive procedure, often performed in selected patients, while biomarkers assessable in blood-derived fluids can be easily translated into routine diagnostic tests.

In this study, we used PEA on OLINK platform to screen 460 different proteins from five panels in serum from PD and PSP patients as well as healthy controls (HC). The most effective proteins in discriminating between the two diseases were identified by ANCOVA, and their classification performance was assessed using a machine learning (ML) technique with XGBoost and random forest algorithms. Finally, the utility of these proteins as diagnostic biomarkers was investigated by ELISA [23], which is largely used in clinical routine.

## 2. Results

Our cohort included 46 PD patients, 30 PSP patients, and 24 HC. Demographic and clinical data of patients and controls are reported in Table 1. While there were no differences in sex, PD patients were slightly younger than PSP and HC. Disease duration was slightly longer in PD than in PSP, but it was not significantly different between the two patient groups. On the other hand, PSP showed higher on the MDS-Unified Parkinson’s Disease Rating Scale (MDS-UPDRS) than PD, reflecting higher disease severity.

### 2.1. Biomarkers Assessment by ANCOVA

**Figure 1:**
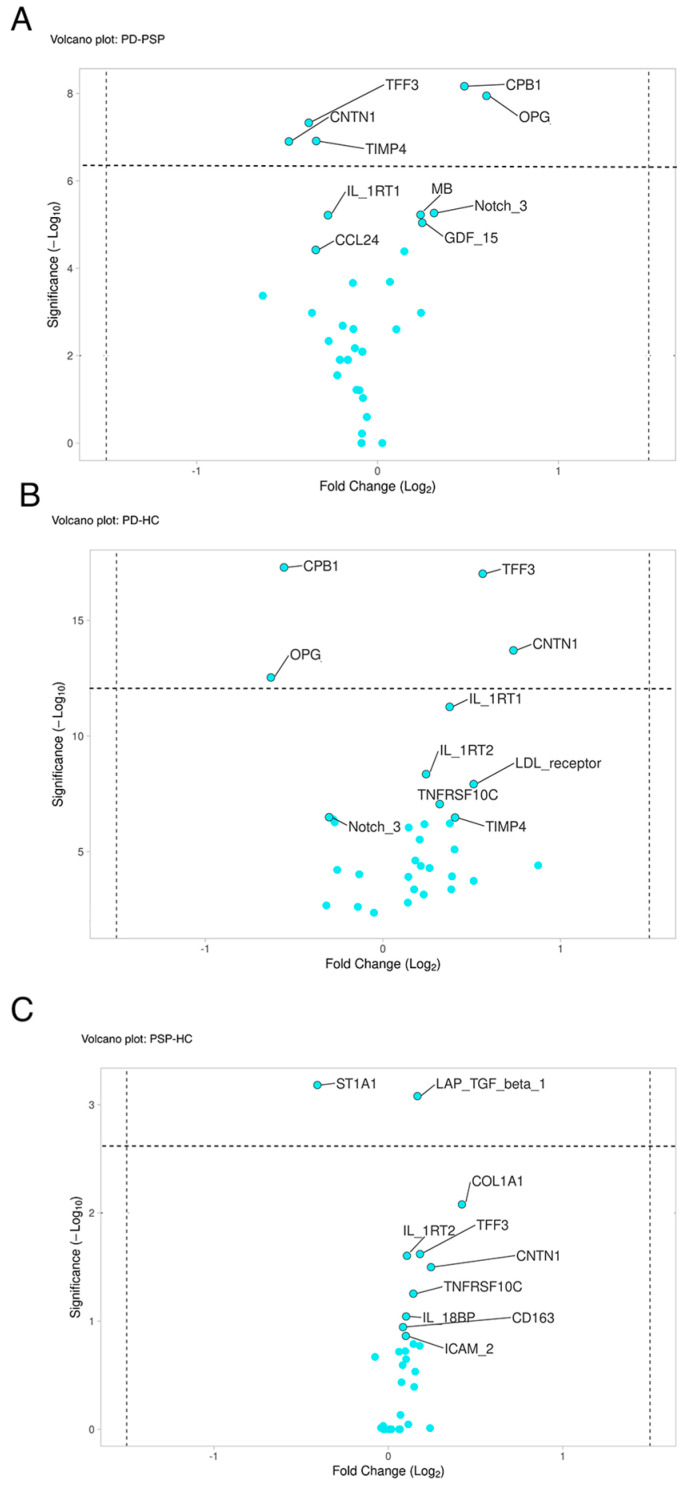
In Panel (**A**), proteins on the right of the *x*-axis have a higher concentration in PSP while proteins on the left have a higher concentration in PD. In Panel (**B**), proteins on the right of the *x*-axis have a higher concentration in PD while proteins on the left have a higher concentration in HC. In Panel (**C**), proteins on the right of the *x*-axis have a higher concentration in HC while proteins on the left have a higher concentration in PSP. Bonferroni’s correction method was employed to define the threshold for *p*-value significance. PD = Parkinson’s disease; PSP = progressive supranuclear palsy; HC = healthy control.

To distinguish betweewn PD, PSP, and HC, an ANCOVA test was performed for each OLINK panel using age, the plate run number and disease duration (where appropriate) as covariates. After the ANCOVA and post hoc tests, proteins not statistically significant for any comparison were removed. Based on this criterion, the final dataset included 78 proteins (7 from Cardiometabolic panel, 58 from Cardiovascular panel, 4 from Immunoresponse panel, 8 from Inflammation panel and 1 from Neuroexploratory panel). In detail, 33 proteins were significant in the comparison PD-HC, 32 proteins were significant in the comparison PD-PSP and 28 proteins were significant in the comparison PSP-HC. A volcano plot for each comparison is shown in Figure 1; the proteins above the dotted line were statistically different between groups after bonferroni correction for the number of tests. Collectively, for the comparison PD-PSP, 5 proteins (TFF3, CPB1, OPG, CNTN1, TIMP4) remained significant (Figure 1A), while 4 (TFF3, CPB1, OPG, CNTN1) and 2 proteins (LAP/TGFβ1, ST1A1) remained significant in the comparison of PD-HC and PSP-HC, respectively (Figure 1B,C), after Bonferroni’s correction. Of note, four proteins (TFF3, CPB1, OPG, CNTN1) were significant in multiple comparisons. The effect size values for each comparison are shown in Table S1. The ANOVA test was repeated employing boostraping procedure, and the results confirmed the high significance of the 7 proteins, all showing the upper limit of the p-value Confidence Intervals under 0.05. Therefore, 7 proteins (TFF3, CPB1, OPG, CNTN1, TIMP4, LAP/TGFβ1, ST1A1) were overall selected and included in further analyses (Table S2).

### 2.2. Classification Performance of ML Models

The ML models were used for three different comparisons, PD-HC, PD-PSP and PSP-HC, using the 7 proteins identified by ANCOVA. The cross-validation performances of XGBoost [24] ML models in each classification task are shown in Table 2. The best performance was obtained in distinguishing between PD and HC (AUC: 0.959 ± 0.029), while the lowest performance was obtained in distinguishing between PSP and HC (AUC: 0.768 ± 0.083). The classification task between PSP and PD reached a good performance with an AUC of 0.892 ± 0.067. Notably, the feature selection procedure identified the TFF3, as the most important feature for distinguishing PD from both PSP and HC, suggesting its possible role in PD, while other proteins were selected as relevant in the comparison between PSP and HC. These results were confirmed using random forest, which showed performance very similar to those obtained with XGBoost (AUC: 0.870 ± 0.065 in PD-PSP, AUC: 0.973 ± 0.026 in PD-HC and AUC: 0.783 ± 0.126 in PSP-HC comparisons).

### 2.3. Biomarkers Validation by ELISA

**Figure 2:**
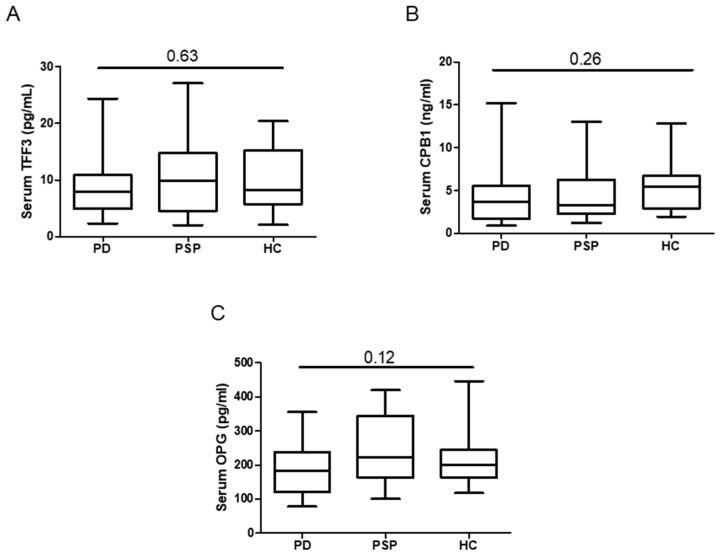
Serum concentration of TFF3 (**A**), CPB1 (**B**), and OPG (**C**) in PD (n = 46), PSP (n = 30) and HC (n = 24), as measured by ELISA. In each box plot, the 25th percentile, 75th percentile, and median of data are depicted as lower, upper, and middle lines, respectively, while bars on vertical lines indicate ranges. The Kruskal–Wallis test was used to calculate shown p-values. TFF3 = trefoil factor 3; CPB1 = carboxypeptidase B1; OPG = osteoprotegerin; PD = Parkinson’s disease; PSP = progressive supranuclear palsy; HC = healthy controls.

To evaluate the utility of OLINK-derived biomarkers in clinical practice, we quantified the most significant proteins for differentiating between PSP and PD (TFF3, CPB1 and OPG; Table S1) by using ELISA, which is a simple and rapid technique widely used in clinical practice. None of the selected biomarkers, however, showed significantly different serum concentration between groups (Figure 2). Similar results were obtained when the analysis was corrected for age and disease duration (Table S3).

### 2.4. Linear Regression

The possible associations between serum biomarkers and demographic or clinical variables in PD (Table 3), PSP (Table 4) and HC (Table 5) were assessed by Linear Regression tests. Serum TFF3 positively correlated with age in all groups, while OPG and CPB1 correlated with age in PSP patients only (Table 3, Table 4 and Table 5). Moreover, serum CPB1 was positively associated with disease severity in PD, correlating with HY Staging Scale (Table 3).

## 3. Discussion

Currently, the clinical diagnosis of PSP relies on the assessment of parkinsonian symptoms along with the presence of additional signs such as slowness of vertical saccades or vertical supranuclear gaze palsy [2]. However, these typical signs may appear late, making early diagnosis challenging [25,26]. Furthermore, the early clinical differentiation between PSP and PD is complicated by the existence of distinct PSP subtypes that vary in progression rates, symptoms and clinical severity, with some subtypes strongly resembling PD [25]. Therefore, intense efforts have focused on identifying easily assessable biomarkers to help clinicians make an accurate diagnosis [6,27]. In this context, blood-based biomarkers are highly preferable over cerebrospinal fluid ones due to the possibility to obtain the biological sample with safe and minimally invasive procedures. Currently, the best peripheral biomarker to discriminate PSP from PD is the neurofilament light chain (Nf-L), an axonal protein that is typically higher in PSP than in PD patients [28,29] and has been proven to be particularly effective as diagnostic biomarker when combined with MRI measurements [30,31]. However, Nf-L is a marker of axonal degeneration, and its serum levels also rise in many other neurodegenerative disorders [28,29], making the identification of more specific diagnostic biomarkers an urgent unmet need.

In this study, we employed for the first time PEA on OLINK platform to conduct an unbiased screen of 460 proteins in serum, aiming to identify peripheral biomarkers to distinguish between PD and PSP patients. Previous studies [16,17,18,19] employed the same technique on blood-derived samples but only investigated proteins differentially expressed between PD and HC without comparing PD and atypical parkinsonism. On the other hand, two other studies focused on biomarkers to distinguish PD from atypical parkinsonisms [20,21], but these data were obtained in CSF samples. Moreover, there are key differences in the experimental settings across studies. In detail, we analyzed five OLINK panels, while previous works assessed one or two panels only [20,21]. In addition, the parkinsonism cohort comprised multiple system atrophy and vascular parkinsonism in one study [20], while it included PSP, cortico-basal syndrome and multiple system atrophy in another report [21]. Overall, such differences make the comparison of the results across studies challenging.

In the current study, we identified five proteins (TFF3, CPB1, OPG, CNTN1, TIMP4) significantly different between PSP and PD patients by using PEA (OLINK) technology, and a combination of these protein levels showed a good performance in discriminating between these two diseases (AUC: 0.892) using a machine learning approach. These high performances were confirmed using two different decision tree-based algorithms (XGBoost and Random Forest), increasing the reliability of this finding. This result is in line with several studies employing machine learning technology to combine several markers for patient classification [32,33]. Notably, 4 out of these 5 proteins (TFF3, CPB1, OPG and CNTN1) also distinguished PD from HC. The importance of OPG protein is in line with a previous work performed by Hepp et al. [19], who also identified this protein as relevant using PEA (Olink) technology.

We further measured by ELISA the three most significant proteins in discriminating between PD and PSP patients (TFF3, CPB1 and OPG) to evaluate their diagnostic value and applicability in clinical practice, where simple easy-to-read assays are desirable. However, none of these proteins differed significantly between groups when measured by ELISA commercial kits. While there are no available previous data on TFF3 or CPB1 assessment by ELISA in Parkinsonian syndromes, conflicting results have been reported on OPG protein, which was found to be increased or reduced in PD compared to HC [34,35]. Here, we did not detect any difference between PD and HC, highlighting the need for further studies on this protein. The discrepancies between results obtained by PEA and ELISA technology could be due to the differences between the two methodologies. Among the proteomic approaches, PEA is considered as the most translatable, since it is an antibody-based assay requiring minimal sample processing before analysis [22]. However, it should be kept in mind that the two methodologies employ different strategies to quantify the analytes. PEA technology uses fluorescence, which is measured over a dark background and allows the detection of very low levels of light [22]. On the other hand, ELISA exploits absorbance, which is measured over a bright background and is less sensible to small differences in light intensity [23]. Accordingly, sensitivity of ELISA is usually in the range of picograms/milliliter (in the assays employed here, sensitivity spares from 1 to 20.5 pg/mL) while PEA sensitivity reaches fentograms/milliliter. Therefore, it is possible that differences in serum biomarkers detected by PEA were too small to be confirmed by ELISA technology. The antibody pairs employed in the two techniques are also different since in PEA, they must recognize epitopes close enough for elongation to occur, while in ELISA, recognized epitopes are usually opposed [22,23]. Regarding specificity, commercial kits such as those used in this study are usually highly specific and do not display particular issues. On the other hand, some cross-reactivity can occur in multiplex ELISA, where multiple antibody pairs are used, reducing specificity [23], but this is not the case of the current study. This limitation is overcome in PEA by conjugating antibody pairs with oligonucleotides containing matched primers annealing only to each other, making PEA technology more suitable for screening dosages of multiple analytes [22]. These differences highlight the need for future development of increasingly sensitive and specific assays to be used in research and clinical settings to address translationability challenges and achieve more reliable results. Nevertheless, PEA results may be validated by ELISA into alternative body fluids or matrices such as neural-derived vesicles, which better represent the central nervous system (CNS) and may be less complex in terms of protein content compared to plasma or serum [36]. Overall, our results suggest that PEA is a reliable and sensitive technique for wide proteomic screening, but the discrepancy between PEA and ELISA dosages detected in the current study suggests caution when translating new discoveries from proteomics into clinical practice, making validation studies with ELISA or SIMOA necessary.

Finally, we identified associations between serum biomarkers and demographic or clinical variables in our cohorts. In detail, we showed that TFF3 serum levels positively correlated with age in all groups, while OPG levels correlated with age in PSP patient only. Moreover, we identified a positive correlation between CPB1 and OPG positively correlated with disease severity in PD patients.

The TFF3 (trefoil factor 3) is a small peptide mainly produced by gut epithelial and goblet cells to maintain the integrity of intestinal barrier. Moreover, TFF3 is expressed also by a variety of different organs and is generally involved in cell migration and tissue repair processes [37]. TFF3 has been detected in several regions of the brain [38,39], where it acts as neuropeptide participating in several processes such as inflammation [40], memory [41] and dementia development [42,43]. Since aging is characterized by the loss of cell function and accumulation of damage [44], the increased levels of TFF3 observed in all groups might be a general compensatory mechanism to restore damaged tissues. A similar relationship with aging could also exist for OPG (osteoprotegerin) and CPB1 (carboxypeptidase B1). OPG belongs to the RANK/RANKL/OPG system, which is a pathway involved in bone remodeling, adaptive immune function and inflammation. While the interaction between RANK and RANKL promotes inflammation, OPG acts as decoy receptor for RANKL, preventing its binding to RANK and thus attenuating pro-inflammatory signals [45]. In the central nervous system, RANKL activates microglia and astrocytes, inducing an inflammatory cascade that would contribute to neurodegeneration, while OPG may modulate such response [46]. On the other hand, CPB1 is a plasmatic enzyme able inactivate pro-inflammatory mediators such as bradykinin, osteopontin and complement components [47]. Since PSP patients typically show a more aggressive phenotype compared to PD [2], the age-related increase of these two proteins could represent further compensatory pathways to counteract a more pronounced inflammatory state [48].

The association of serum CPB1 levels with severity in PD might be related to widespread deposition of α-synuclein occurring not only within the brain but also in peripheral districts [49]. Such inclusions have been demonstrated to promote complement activation with cytotoxic effects [50]. Therefore, the CPB1 elevation in PD might represent a mechanism to limit systemic inflammation causing progressive neuronal damage. In summary, CPB1 could represent a useful biomarker to support clinical staging of PD, although further research in larger cohorts is warranted to confirm such association and to clarify the underlying molecular mechanisms.

Our study has some limitations to be considered. First, the number of subjects per group is relatively small, although the sample size of PSP is in line with most studies in the field due to the low incidence of the disease; this limitation did not allow deep patient stratification according to disease severity or subtype. Second, this is a pilot study lacking an external validation cohort to confirm the results. This implies that our results need to be confirmed in larger studies to demonstrate the generalizability of the findings. Third, patients’ diagnoses were made by movement disorder specialists with more than 10 years of experience based on international criteria [1,2] but lacked histopathological confirmation, and some misdiagnosis might have occurred.

## 4. Materials and Methods

### 4.1. Patients

In total, 46 PD patients, 30 PSP patients, and 24 healthy controls were enrolled in this study. Clinical diagnosis was established according to the recent MDS diagnostic criteria for PD and PSP [1,2]. Of the 30 PSP patients, 23 had probable PSP-Richardson’s Syndrome (PSP-RS) and 7 had probable PSP-Parkinsonism (PSP-P) [2]. All patients underwent a neurological examination in practical OFF performed by the same movement disorder specialist, including MDS-UPDRS [51] and HY rating scales, and PSP patients were further evaluated by using PSP rating scale [52]. In addition, patients underwent a 3T brain MRI scan with a recently described protocol [53,54]. Exclusion criteria for patients consisted of clinical features suggestive of other diseases, MRI abnormalities such as neoplasia, signs suggestive of multiple system atrophy or normal pressure hydrocephalus, and lacunar infarctions in the basal ganglia or diffuse subcortical vascular lesions. None of the control participants had a history of neurologic, psychiatric, or other major medical illnesses as well as no close relatives with neurodegenerative diseases. All the study procedures and ethical aspects were in accordance with the Declaration of Helsinki and approved by the Calabria Region Ethics Committee. All the subjects involved gave written informed consent for participation in the study and the use of their medical records for research purposes.

### 4.2. Serum Sample Collection

Blood was collected from each subject between 9 a.m. and 12 p.m. in BD Vacutainer^TM^ SST^TM^ Serum Separation Tubes (BD, Franklin Lakes, NJ, USA) and processed within 30 min after collection. Serum was obtained by centrifuging tubes at 3000 rpm at 4 °C for 10 min. Then, each sample was aliquoted and stored at −20 °C until use. For biomarkers assessment, aliquots were thawed at 4 °C few hours before the assay and vortexed thoroughly. Each serum aliquot was used once.

### 4.3. Proximity Extension Assay by Olink

Serum biomarker testing was performed by PEA technology on OLINK platform (Olink, Uppsala, Sweden) [22]. We run five protein panels: Cardiometabolic, Cardiovascular Immunoresponse, Inflammation and Neuroexploratory, for an overall assessment of 460 proteins. Each panel is composed of 92 biomarkers, whose complete list is available on the company website (https://olink.com/products/olink-target-96 accessed on 9 September 2024). PEA identifies target proteins through polyclonal antibodies labeled with DNA oligonucelotides; when two antibodies bind the same target, a proximity-dependent DNA elongation occurs and target levels are read out by qPCR.

Serum samples from the three subject groups were randomized and loaded into different plates (MicroAmp^®^ Fast Optical 96-well Reaction Plate-Applied Biosystems, Waltham, MA, USA) and processed following the manufacturer’s instructions. In addition, plate controls, negative controls, and sample controls provided by Olink^®^ Proteomics were added to each plate. Negative controls are exclusively composed of buffer to establish background noise and define the limit of detection (LOD), while sample controls are external samples used for the determination of inter- and intra-plate quality of each assay.

Data for each biomarker expression are provided as normalized protein expression (NPX) values, an arbitrary unit on a Log2 scale after internal intensity normalization. NPX values are calculated from the threshold cycle, which is the number of qPCR cycles needed to overcome a fluorescence signal threshold. NPX are proportional to the concentration of the protein (https://olink.com/knowledge/documents accessed on 9 September 2024). We subsequently performed intensity normalization of NPX values to allow the combination of data from different plates, as performed in previous studies [21] and described in the manufacturer manual. Proteins with values below LOD in more than 10% of samples were excluded from the analyses.

### 4.4. Enzyme-Linked Immunosorbent Assay

Serum TFF3, CPB1, and OPG were evaluated by specific ELISA kits. Human TFF3 Quantikine ELISA kit (DTFF30) was provided by R&D Systems (Minneapolis, MN, USA) while human CPB1 and OPG ELISA 96-assays (EH67RB and EHTNFRSF11B, respectively) were provided by Thermo Fisher Scientific (Waltham, MA, USA). For all the assays, the manufacturer instructions were followed. All the measurements were performed in duplicates and plates were read on Thermo Fisher Varioskan™ LUX multimode microplate reader.

### 4.5. Statistical Analysis

Differences in sex distribution were investigated using Fisher’s exact test. The Shapiro–Wilk test was used to check data normality. Based on the distribution, differences in age at examination and ELISA data were assessed using ANOVA or the Kruskal–Wallis test followed by Bonferroni correction, while differences in disease duration, MDS-UPDRS, and HY scores between the two patient groups were assessed using the Mann–Whitney test. The ELISA data were further analyzed through ANCOVA using age and disease duration as covariates. Linear regression analysis was used to investigate the associations between serum biomarkers and demographical or clinical variables. All tests were two-tailed, and the α level was set at *p* < 0.05. Statistical analysis was conducted with R language v.4.1.2 and IBM SPSS v29.0.1.0.

For OLINK data, ANCOVA test with age, disease duration and the plate run as covariates was performed for each panel, followed by post-hoc test in the three different comparisons (PD-HC, PSP-HC, PD-PSP). For each comparison, a volcano plot was built by using the mean and the *p*-value of each protein to visually highlight significantly different proteins. The effect size for each comparison was calculated using Cohen D statistics. To improve the robustness of the procedure we also performed an ANOVA with boostraping to confirm statistical significance.

### 4.6. Machine Learning Approach

In this study, we employed two different machine learning (ML) algorithms, termed XGBoost [24] and random forest (RF) [55], using the seven proteins extracted from the Volcano plots. RF was used to confirm the performance of XGBoost. In the XGBoost model, hyperparameters were tuned using five-fold cross-validation (CV) combined with a randomized search over ten iterations, with the goal of maximizing classification accuracy and reducing overfitting. Briefly, the dataset was split into K subsets (folds), and the model was trained iteratively K times. For each iteration, the model was trained on (K-1) folds and evaluated on the remaining Kth fold, which was left out of the training phase. The hyperparameters tuned for XGBoost included: learning rate, maximum depth, minimum child weight, gamma, and colsample bytree (which is the proportion of features randomly selected to train each tree). For each iteration, a different set of hyperparameters was tested. For each set of hyperparameters, five-fold cross-validation was executed and the average performance metrics were calculated. The set of hyperparameters with the best average performance was identified as the optimal one. Feature importance was assessed using the “permutation accuracy importance” method, which measured the mean decrease in accuracy after permuting each feature, with 50 repetitions to ensure robust feature ranking. Following this, a feature selection process was carried out by iteratively training models using features ranked by their importance; details about feature importance are shown in Supplementary Materials (S1). Finally, the performance of the optimized XGBoost model [24], trained on the most significant features, was evaluated using five-fold cross-validation, calculating the mean and standard deviation of the area under the curve (AUC), accuracy, sensitivity, and specificity across validation folds. A model was deemed capable of distinguishing between groups if the mean AUC exceeded 0.85. The analyses were performed using Python 3.9 and the scikit-learn package version 1.0.2.

## 5. Conclusions

In the current study, we first applied PEA technology to easily accessible blood-derived biofluids to identify protein biomarkers useful for distinguishing PSP from PD patients. We identified a panel of proteins which provided good classification performances through machine learning technology, and we identified a new relevant protein, (CPB1) associated with disease severity in PD patients, which may support clinical staging in PD. Additionally, we compared PEA and ELISA technologies and we found discrepancies between these two techniques, suggesting caution when translating proteomic findings into clinical practice. Proteomic techniques indeed represent powerful strategies for biomarker discovery due to their ability to unbiasedly screen a huge number of proteins, but validation studies using standard technologies for protein dosages, such as ELISA, are warranted before translating proteomic findings into clinical practice.

## Figures and Tables

**Table 1:** Demographic and clinical data of patients with Parkinson’s disease and progressive supranuclear as well as healthy controls.

	PD (n = 46)	PSP (n = 30)	HC (n = 24)	*p*-Value
Sex (F/M)	28/19	21/22	14/17	0.77 ^a^
Age at examination (years)	65.0 ± 8.62 *°	71.0 ± 6.42	70.5 ± 6.00	*0.001* ^b^
Disease duration (years)	6.7 ± 5.87	3.8 ± 2.38	-	0.10 ^c^
MDS-UPDRS	28.6 ± 16.92	44.3 ± 16.99	-	*<0.001* ^c^
PSP rating scale	-	44.0 ± 16.30	-	-

Data are shown as mean ± SD. Abbreviations: PD: Parkinson’s disease; PSP: progressive supranuclear palsy; HC: healthy control; MDS-UPDRS: MDS-Unified Parkinson’s Disease Rating Scale; HY: Hoehn and Yahr. * PD vs. PSP (*p*-value: 0.003). ° PD vs. PSP (*p*-value: 0.13). ^a^ Fisher’s exact test. ^b^ ANOVA followed by Bonferroni’s correction. ^c^ Mann–Whitney test.

**Table 2:** Classification performances and feature selection with XGBoost in distinguishing between PD, PSP and HC.

Feature Selection	PD-PSP	PD-HC	PSP-HC
	AUC: 0.892 ± 0.067	AUC: 0.959 ± 0.029	AUC: 0.768 ± 0.083
	ACC: 0.874 ± 0.051	ACC: 0.943 ± 0.034	ACC: 0.789 ± 0.069
	SENS: 0.8 ± 0.141	SENS: 0.913 ± 0.051	SENS: 0.964 ± 0.041
	SPEC: 0.922 ± 0.054	SPEC: 0.98 ± 0.041	SPEC: 0.45 ± 0.241
Best features	#5	#1	#2
	TFF3	TFF3	LAP/TGFβ1
	CPB1		ST1A1
	OPG		
	CNTN1		
	TIMP4		

**Table 3:** Linear regression analysis between serum protein levels, demographic variables, and clinical variables in PD patients.

PD (n = 46)			**Age**	**Disease** **Duration**	**MDS-** **UPDRS**	**HY Staging** **Scale**
	TFF3	Coefficients	0.42	0.01	0.11	0.14
		*p*-value	*0.003*	0.78	0.41	0.09
	CPB1	Coefficients	0.18	0.09	0.1	0.30
		*p*-value	0.13	0.37	0.55	*0.027*
	OPG	Coefficients	0.16	0.31	0.21	0.37
		*p*-value	0.64	0.968	0.45	0.08

PD: Parkinson’s disease; MDS-UPDRS: MDS-Unified Parkinson’s Disease Rating Scale; HY: Hoehn and Yahr; TFF3: trefoil factor 3; CPB1: carboxypeptidase B1; OPG: osteoprotegerin. Clinical variables were correlated with age as covariate.

**Table 4:** Linear regression analysis between serum protein levels, demographic and clinical variables in PSP patients.

PSP (n = 30)			**Age**	**Disease** **Duration**	**PSP** **Rating Scale**
	TFF3	Coefficients	0.15	0.13	0.13
		*p*-value	*0.001*	0.23	0.51
	CPB1	Coefficients	0.35	0.1	0.1
		*p*-value	*0.01*	0.76	0.67
	OPG	Coefficients	0.42	0.01	0.10
		*p*-value	*0.009*	0.88	0.98

PSP: progressive supranuclear palsy; TFF3: trefoil factor 3; CPB1: carboxypeptidase B1; OPG: osteoprotegerin.

**Table 5:** Linear regression analysis between serum protein levels and age in HC.

HC (n = 24)			**Age**
	TFF3	Coefficients	0.57
		*p*-value	*0.003*
	CPB1	Coefficients	0.10
		*p*-value	0.94
	OPG	Coefficients	0.46
		*p*-value	0.20

HC: healthy control; Yahr; TFF3: trefoil factor 3; CPB1: carboxypeptidase B1; OPG: osteoprotegerin.

## Data Availability

Raw data on protein expression measured by OLINK and ELISA are available as Table S4 in Supplementary Materials.

## Supplementary Materials

The following supporting information can be downloaded at: https://www.mdpi.com/article/10.3390/ijms252111663/s1.
