# Supplementary material for: Proximity Elongation Assay and ELISA for the Identification of Serum Diagnostic Biomarkers in Parkinson’s Disease and Progressive Supranuclear Palsy
Source: Int J Mol Sci. 2024 Oct 30;25(21):11663. doi: 10.3390/ijms252111663 (PMC11546529; doi:10.3390/ijms252111663)
Supplement: Supplementary file 1 [file ijms-25-11663-s001.zip › Supplementary materials_v2.pdf]

Supplementary Methods S1:

The Permutation Importance method was used to calculate feature importance in a non-parametric way by randomly shuffling the values of each feature and observing the impact on the model's performance. This method helps reduce overfitting because it assesses feature importance across different permutations of the data. This ensures that the importance of features is not overly dependent on a specific dataset structure. Cross-validation was employed at each step of feature selection (using cv= 5) to evaluate the model's performance across different data splits. This ensures that the model's performance is not just optimized for one training set, but rather generalizes well across multiple splits. By averaging the performance across folds, we minimize the risk of selecting features that might be overfit to a specific subset of the data. In each iteration, a subset of the most important features was selected, and the model was re-evaluated using cross-validation to compute performance metrics like accuracy, sensitivity, specificity, and AUC. This iterative process of gradually adding features and evaluating model performance on different validation sets further helps control overfitting by selecting only the features that consistently contribute to improving performance across all data splits.

Table S1. Effect size calculated as Cohen d, for each comparison.

	**PSP-PD comparison**	**PD-HC comparison**	**PSP-HC comparison**
TFF3	1.74 (Large)	3.14 (Large)	0.72 (Medium)
CPB1	1.73 (Large)	2.62 (Large)	0.47 (Small)
OPG	1.91 (Large)	2.71 (Large)	0.18 (Small)
CNTN1	1.75 (Large)	3.22 (Large)	0.70 (Medium)
TIMP4	1.57 (Large)	1.81 (Large)	0.24 (Small)
LAP/TGFβ1	0.20 (Small)	1.08 (Large)	1.15 (Large)
ST1A1	0.21 (Small)	0.96 (Large)	1.06 (Large)

Abbreviations: PD: Parkinson's disease; PSP: progressive supranuclear palsy; HC: healthy control

Table S2. NPX values of the significant 7 proteins in patients with PD and PSP as well as in HC. Data are shown as mean ± SD.

	PD (n = 46)	PSP (n = 30)	HC (n = 24)	p-value
TFF3	6.44 ± 0.68°#	4.77 ± 1.01	4.15 ± 0.59	5.35e-21[b]
CPB1	5.85 ± 1.18°#	8.06 ± 1.39	8.62 ± 0.70	3.34e-15[b]
OPG	3.27 ± 0.62°#	4.66 ± 1.04	4.84 ± 0.66	3.08e-13[b]
CNTN1	4.14 ± 0.47°#	2.98 ± 0.84	2.45 ± 0.57	7.45e-17[b]
TIMP4	4.77 ± 0.57°#	3.65 ± 0.71	3.47 ± 0.77	1.97e-12[b]
LAP/TGFβ1	8.00 ± 0.77	8.43 ± 0.88+	7.49 ± 0.85	6.18e-5[b]
ST1A1	4.80 ± 0.82	5.45 ± 0.94+	4.10 ± 1.32	1.11e-5[b]

PD: Parkinson's disease; PSP: progressive supranuclear palsy; HC: healthy control

° PD vs. PSP (p-value < 0.05)

PD vs. HC (p-value < 0.05)

+ PSP vs. HC(p-value < 0.05)

[b] ANCOVA with Bonferroni's correction with age, disease duration and number of run as covariates.

Table S3. Differences in serum concentration of the 3 most significant proteins in patients with PD and PSP patients as well as in HC.

	PD (n = 46)	PSP (n = 30)	HC (n = 24)	p-value
TFF3	9.6 ± 0.82	8.6 ± 1.09	9.4 ± 1.02	0.50[b]
CPB1	4.4 ± 0.67	4.4 ± 0.80	5.4 ± 0.65	0.99[b]
OPG	198.2 ± 14.69	229.5 ± 18.33	206.9 ± 15.77	0.22[b]

Data are shown as mean ± SD and corrected for age and disease duration

PD: Parkinson's disease; PSP: progressive supranuclear palsy; HC: healthy control

[b] ANCOVA with age and disease duration as covariates.
